# Tolerance and Excretion of the Mycotoxins Aflatoxin B_1_, Zearalenone, Deoxynivalenol, and Ochratoxin A by *Alphitobius diaperinus* and *Hermetia illucens* from Contaminated Substrates

**DOI:** 10.3390/toxins10020091

**Published:** 2018-02-24

**Authors:** Louise Camenzuli, Ruud Van Dam, Theo de Rijk, Rob Andriessen, Jeroen Van Schelt, H. J. (Ine) Van der Fels-Klerx

**Affiliations:** 1 ExxonMobil Petroleum & Chemical, Hermeslaan 2, 1831 MAchelen, Belgium; louise.camenzuli@exxonmobil.com; 2 RIKILT Wageningen Research, Akkermaalsbos 2, 6708 WB Wageningen, The Netherlands; ruud.vandam@wur.nl (R.V.D.); theo.derijk@wur.nl (T.d.R.); 3 Proti-Farm, Harderwijkerweg 141a, 3852 AB Ermelo, The Netherlands; randriessen@protifarm.com; 4 Koppert BV, Veilingweg 14, 2650 AD Berkel en Rodenrijs, The Netherlands; JvSchelt@Koppert.nl

**Keywords:** insects, food safety, feed safety, contaminants, bioaccumulation, excretion, lesser mealworm, black soldier fly, *Alphitobius diaperinus*, *Hermetia illucens*

## Abstract

This study aimed to investigate the potential accumulation of mycotoxins in the lesser mealworm (*Alphitobius diaperinus,* LMW) and black soldier fly (*Hermetia illucens,* BSF) larvae. Feed was spiked with aflatoxin B_1_, deoxynivalenol (DON), ochratoxin A or zearalenone, and as a mixture of mycotoxins, to concentrations of 1, 10, and 25 times the maximum limits set by the European Commission for complete feed. This maximum limit is 0.02 mg/kg for aflatoxin B_1_, 5 mg/kg for DON, 0.5 mg/kg for zearalenone and 0.1 mg/kg for ochratoxin A. The mycotoxins and some of their metabolites were analysed in the larvae and residual material using a validated and accredited LC-MS/MS-based method. Metabolites considered were aflatoxicol, aflatoxin P_1_, aflatoxin Q_1_, and aflatoxin M_1_, 3-acetyl-DON, 15-acetyl-DON and DON-3-glycoside, and α- and β-zearalenol. No differences were observed between larvae reared on mycotoxins individually or as a mixture with regards to both larvae development and mycotoxin accumulation/excretion. None of the mycotoxins accumulated in the larvae and were only detected in BSF larvae several orders of magnitude lower than the concentration in feed. Mass balance calculations showed that BSF and LMW larvae metabolized the four mycotoxins to different extents. Metabolites accounted for minimal amounts of the mass balance, except for zearalenone metabolites in the BSF treatments, which accounted for an average maximum of 86% of the overall mass balance. Both insect species showed to excrete or metabolize the four mycotoxins present in their feed. Hence, safe limits for these mycotoxins in substrates to be used for these two insect species possibly could be higher than for production animals. However, additional analytical and toxicological research to fully understand the safe limits of mycotoxins in insect feed, and thus the safety of the insects, is required.

## 1. Introduction

Nowadays, insects are considered a promising alternative protein source for use in feed and food applications in Europe. In particular, larvae of *Alphitobius diaperinus* (lesser mealworms, LMW) and *Hermetia illucens* (black soldier flies, BSF) are considered a sustainable source of high-quality protein and certain vitamins and minerals and are amongst the insect species gaining most interest for feed and food applications [1]. While national policies in several European countries already permit the use of certain insect species in food applications, an overall European policy is still under development. One of the main topics in this process of regulating the use of insects in feed and food is ensuring the chemical safety of the insects for animal and human consumption.

Within Europe, maximum limits (ML) have been set for the presence of various chemical contaminants in a range of animal feeds, human food products and ingredients [2]. These include, amongst others, ML for the presence of the mycotoxin aflatoxin B_1_ (AfB1) in animal feed [3] and human food products [2], and guidance levels for several other mycotoxins, including deoxynivalenol (DON), zearalenone (ZEN), ochratoxin A (OTA), and fumonisin B_1_/B_2_, T-2/HT-2 toxins, in animal feed [4]. When insects are used for feed and food, they should comply with these regulations. However, to date, it is often not completely clear if insects are within the scope of the regulation since they are not specifically mentioned.

In its opinion on the safety of insects for use in feed and food in 2015, the European Food Safety Authority (EFSA) concluded that scientific data on the safety of insects are lacking [5]. Since then, several studies have been performed and published investigating the potential contamination of insects with food safety hazards, mainly focusing on chemical contaminants. These include, amongst others, heavy metals [6,7], pesticides [8], DON [9], and AfB1 [10]. Both the two latter studies showed that the DON and AfB1 did not accumulate in the insect species studied. Bosch et al. [10] suggested that BSF metabolize AfB1 and concluded that further research into potential metabolites was warranted. Furthermore, since mycotoxins often co-occur, they suggested to study the tolerance and accumulation of individual and mixtures of mycotoxins in selected insect species. Furthermore, controlled experiments, like performed with DON and AfB1 [9,10], have not been done yet for other mycotoxins.

The aim of this study was to investigate the potential accumulation of four different mycotoxins in larvae of the LMW and the BSF, using feed artificially contaminated at three different doses. The insects were reared on feed spiked with the mycotoxins individually and also as a mixture in order to determine the effect of exposure to co-occurring mycotoxins. These two species were chosen because they have been identified as being among the most interesting insect species for large-scale production for use as feed and food materials [5,11]. Mycotoxin contamination levels were chosen at levels relative to the respective limits set by the European Commission (EC limit) for complete feed such to investigate whether these current European Commission limits are suitable for these two insect species.

## 2. Results

### 2.1. Mycotoxin Concentration in Feed

Concentrations of AfB1, DON, ZEN, and OTA in the original (non-spiked) feed used for insect rearing were below the limit of quantification (LOQ) for the respective mycotoxins. Given the absence of these mycotoxins, the metabolites considered in this study were also assumed to be absent, and hence the control feed was considered to be “mycotoxin-free”. The average mycotoxin concentration in the feed spiked with individual mycotoxins separately varied from 33 to 170% of the intended spiked concentration being the 1× the EC ML (L1), 10× the EC ML (L2) and 25× the EC ML (L3) (Table 1). For mixtures the quantified concentrations in the feed varied between 75% and 90% of the intended spiked concentration (Table 1). The intended concentration ratios between L1:L2 and L1:L3 were respected for the feed spiked with the mycotoxin mixture. However, for the individual treatments, L1 was 8–48 times lower than the respective L3 treatment. Non-homogeneously mixed feed was ruled out, since homogeneity test performed on the M1 treatment confirmed a homogenously distributed sample (relative standard deviation: 5.2–6.1%). It is not clear what could have caused the deviations between intended and observed spiked concentrations is not clear. Since the true concentrations are known, the spiked samples were deemed fit for the experiment.

### 2.2. Effect of Mycotoxins on Insect Development

**Figure 1:**
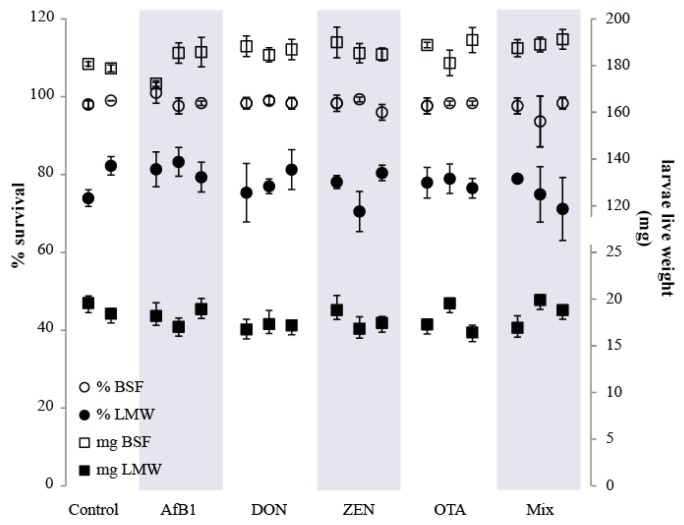
Mean percentage survival including the standard deviation (*n* = 3) of the black soldier fly (BSF) (o) and the lesser mealworm (LMW) (●), and the mean larvae live weight at the end of the treatment including the standard deviation (*n* = 3) of the BSF (□) and the LMW (■) reared on mycotoxin-free feed (control), and feed spiked to three levels of AfB1, DON, ZEN, and OTA (L1–L3, left to right), and on feed spiked with a mixture of all four mycotoxins (M1–M3, left to right) as indicated in Table 1.

The average survival rate of the BSF larvae after 12 days ranged from 94 to 100% (*n* = 17 treatment sets) (Figure 1). In one single M2 treatment, a survival rate of 104% was observed, which could be due to a miscount at the beginning of the experiment (when larvae are really small), and this survival rate was set to 100%. No difference in the survival rate was observed between the larvae reared on control feed and the larvae reared on mycotoxin contaminated feed (*p* > 0.05). Additionally, survival rate was not different between the different treatments (single and mix; *p* > 0.05). The average wet live weight of the BSF (average per treatment set) ranged from 172 ± 1 mg/larvae (AfB1-L1 treatment) to 191 ± 4 mg/larvae (M3 treatment), with no significant difference observed amongst all treatments (*p* > 0.05). These results imply that the rearing of BSF on feed contaminated with individual mycotoxins, or a mixture of mycotoxins does not impact the development of the larvae.

The average survival rate of the LMW larvae after 16 days ranged from 71 to 83% between the 17 treatment sets. No significant differences in the average survival rates amongst the various treatments were observed (*p* > 0.05). The average wet live weight of the LMW larvae ranged from 16.4 ± 0.8 mg/larvae (OTA-L3) to 19.9 ± 0.5 mg/larvae (M2). There was no significant difference (*p* > 0.05) in the average live weights among most of the various treatments compared to the controls. The larvae reared on OTA-L3 had an average live weight significantly lower (*p* = 0.03) than the larvae reared on the control feed, as well as significantly lower weight (*p* = 0.03) than larvae from treatment OTA-L2. Larvae from treatment M2 had an average live weight significantly higher than the larvae from treatments DON-L1, ZEN-L2 and OTA-L3. All results are available in Table S1 of the Supplementary Materials.

### 2.3. Mycotoxin Accumulation in Insects

**Figure 2:**
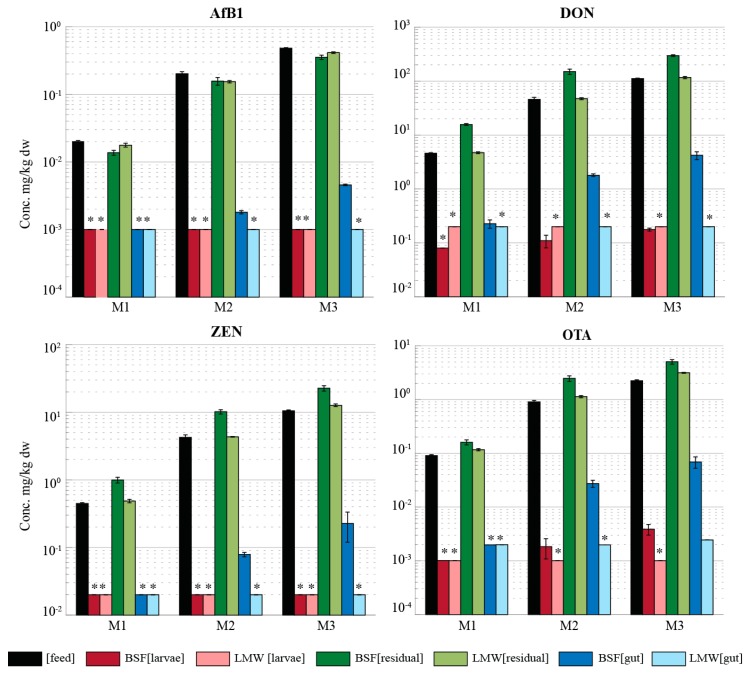
Concentrations (mg/kg dw ± standard deviation) of AfB1, DON, OTA, and ZEN in feed (**black**), larvae (**red**), residual material (spiked feed) (**green**), and residual material (gut clean) (**blue**) in black soldier fly (BSF) and lesser mealworm (LMW) of mixture treatments exposed to three mycotoxin levels (M1–M3). * in the figure indicates that concentration is below the LOQ, and the LOQ is plotted in the figure. Residual material (gut clean) refers to the residual materials after given the insects clean feed for two days.

Figure 2 shows the mycotoxin concentrations measured in the feed, larvae, amd residual material (RM) from the period when the insects were reared on spiked feed—RM (spiked feed) and residual material from the period when the insects were put on clean feed—RM (gut clean)—for both BSF and LMW larvae reared on the mixture of the four mycotoxins at three intended spiked levels (M1–M3). The results of the larvae reared on the individual mycotoxins separately (L1–L3) follow a similar pattern to the results of the larvae reared on the mixture of the mycotoxins and hence are not included in the figure (all results are available in Table S2 of the Supplementary Materials). No accumulation of AfB1 was observed in BSF larvae, even when reared on feed spiked with a mixture of mycotoxins up to about 25 times the limit set by the European Commission for the respective mycotoxin in feed (M3) (Figure 2). DON, ZEN, and OTA were detected in the BSF larvae above the LOQ. However, the concentrations were several orders of magnitude lower than the respective concentration in their feed. The concentrations of all mycotoxins in the LMW larvae were below their respective LOQ’s in all the individual and mixture treatments (light red bars Figure 2).

The mycotoxin concentrations in the RM (spiked feed) is shown in Figure 2 as green bars (dark green: BSF and light green: LMW). The increasing mycotoxin concentration in the spiked feed (black bars) from M1 to M3 is consistent with the increasing concentration in the RM (spiked feed) of both insects for the respective treatment (Figure 2). However, AfB1 concentration in the RM (spiked feed) of both insects is lower than the AfB1 concentration spiked in the feed for the three levels. The highest AfB1 concentration in the RM (spiked feed) was measured in the M3 at levels of 380 µg/kg_dw_ and 420 µg/kg_dw_ for the BSF and LMW larvae, respectively. For all treatments, the average AfB1 concentration in the RM (spiked feed) was 0.81 (±0.11) and 0.88 (±0.11) times lower than the AfB1 concentration in feed for the BSF and the LMW larvae, respectively. For both insect species, no clear difference could be observed amongst the treatments on the ratio of AfB1 in feed and in the RM (spiked feed). This was true for both individual treatment and the mixtures and also between the different levels.

While no differences were observed between the ratios of the AfB1 concentration in the RM (spiked feed) and in the feed for both insects, this was not the case for the other three mycotoxins. The DON concentration in the RM (spiked feed) of the BSF larvae was on average 2.4 (±0.65) and 3.5 (±0.49) times higher than the respective spiked concentration in feed for the individual and mixture treatments, respectively. Similar results were observed for ZEN (2.6 ± 0.20 and 2.5 ± 0.23) and OTA (2.7 ± 0.66 and 2.5 ± 0.52) treatments of the BSF larvae reared on feed spiked with individual and mixture mycotoxins respectively. Conversely, the DON, ZEN, and OTA concentrations in the RM (spiked feed) of the LMW larvae was generally not different from the concentration in feed, with ratios of residual material to feed very close to 1.

The mycotoxin concentration in the RM (gut clean) is shown in Figure 2 as blue bars (dark blue: BSF and light blue: LMW). AfB1, DON, ZEN, and OTA were detected in the RM (gut clean) of the BSF larvae in every treatment except for AfB1 and ZEN in the L1 treatments in both the individual and mixture treatments (Figure 2). The mycotoxin concentrations were several orders of magnitude lower compared to the concentration in the RM (spiked feed). The maximum AfB1 concentration in the RM (gut clean) of the BSF was 6.4 µg/kg_dw_ (L3), which is 50 times lower than the AfB1 concentration in the RM (spiked feed) and 60 times lower than the AfB1 concentration in the feed. The highest DON concentration in the RM (gut clean) of the BSF was 6.8 mg/kg_dw_ in the L3 treatment, which is 17 times lower than the concentration in the spiked feed. For the LMW larvae, concentrations of AfB1 and ZEN in the RM (gut clean) were all below the respective LOQ (<1 µg/kg_dw_ and <20 µg/kg_dw_, respectively). However, DON was detected in the RM (gut clean) of the L3 treatment with a mean concentration of 263 µg/kg_dw_ (430 times lower than the concentration in the feed) (Table S2 in Supplementary Materials). OTA was detected in the RM (gut clean) M3 (mean concentration 2.4 µg/kg_dw_) and in one treatment of the M2 (2.7 µg/kg_dw_). The measured concentrations of DON and OTA were slightly above the LOQ (200 µg/kg_dw_ and 2 µg/kg_dw_ respectively), and hence the slight differences between the measurements in the RM (gut clean) for the individual and mixture treatments was due to the different feed concentrations.

### 2.4. Mycotoxin Metabolites

In the BSF larvae, the presence of all measured aflatoxin metabolites (aflatoxicol, aflatoxin P_1_, aflatoxin Q_1_, and aflatoxin M_1_) and all measured DON metabolites (15- & 3-Acetyl-DON and DON-3-glucoside) were below their respective LOQs regardless of the treatments. Quantifiable concentrations of ZEN metabolites (α- & β-zearalenol) were only found in the L2, L3, M2, and M3 treatments of the BSF (Table 2). In the LMW larvae, all metabolites analysed were below the LOQs (Table 2). The LOQs for aflatoxin Q_1_, 3-Acetyl-DON and DON-3-glucoside in the LMW larvae were higher than for the other metabolites due to matrix interference observed during the LC-MS/MS analyses.

In the RM (spiked feed), aflatoxicol was only detected in the higher treatment levels (L3 and/or M3) for both insects, while aflatoxin M_1_ (AfM1) was only detected at the two highest treatment levels (L2, L3, M2, and M3) for the LMW but not for the BSF (Table 2). A signal above the LOQ for aflatoxin P_1_ was detected in the L2, L3, M2, and M3 of the BSF, but the concentrations could not be determined due to matrix interferences. No other AfB1 metabolites were detected in the RM (spiked feed) of the BSF and LMW. For the RM (gut clean), all monitored aflatoxin metabolites were below their respective LOQ for all treatments of both BSF and LMW. All DON metabolites were below their LOQ in the RM (spiked feed), as well as in the RM (gut clean) of all treatments of both insects.

Both α- and β-zearalenol were detected in the RM (spiked feed) of all treatments for both insects, except for the β-metabolite in the individual L1 treatment of the LMW (Table 2). While BSF and LMW larvae were reared on the same spiked feed, the concentrations of α- and β-zearalenol were up to 50 and 40 times higher, respectively, in the RM (spiked feed) of the BSF as compared to LMW. The maximum mean concentration of the α-metabolite in the RM (spiked feed) of the BSF was 37.3 mg/kg_dw_ (L3 treatment), a concentration that is four times lower than the mean concentration of ZEN in the same treatment.

### 2.5. Mass Balance

Mass balance calculations were performed to investigate how much of the original amount of mycotoxin given to the insects (with spiked feed) was found back in the larvae and the two types of residual materials. The calculations were based on the concentration of the mycotoxins in the spiked feed on which the larvae were reared in relation to the quantified concentrations of the mycotoxin and its metabolites in larvae, RM (spiked feed), and RM (gut clean). All measurements of mycotoxins and metabolites below their respective LOQs were set to zero in these samples for the mass balance calculations. Setting measurements for the metabolites below LOQ to half the LOQ contributed minimally (generally below 1%) to the overall mass balance. 

**Figure 3:**
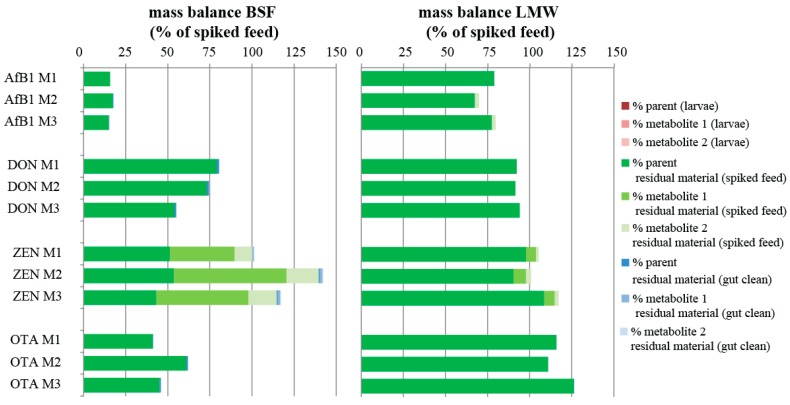
Mass balance of aflatoxin B_1_, deoxynivalenol, zearalenone, and ochratoxin A in black soldier fly (BSF) and lesser mealworm (LMW) experiment. Mass balance calculations include metabolites measured above the limit of quantification. See Table 2 for detected aflatoxin B_1_ (metabolite 1 = aflatoxicol; metabolite 2 = aflatoxin M_1_) and zearalenone (metabolite 1 = α-zearalenol; metabolite 2 = β-zearalenol) metabolites. M1–M3 refer to spiked levels presented in Table 1. Mass balance was calculated—per mycotoxin—as: (sum of the amount of mycotoxin and its metabolites in the larvae and both residual materials/amount of mycotoxin in the feed) * 100%.

The average mass balance for AfB1 in the BSF ranged between 11% and 18% among the different treatments (Figure 3 and Table S3 of the Supplementary Materials). Aflatoxicol, the only metabolite detected in the BSF (Table 2), accounted for 0.2% of the overall mass balance of AfB1 in the BSF. In the LMW, between 56% and 80% of the amount of AfB1 the insects were exposed to could be accounted for. Aflatoxicol and AfM1 detected in the RM (spiked feed) accounted for a maximum of 0.3 and 2.5% of the total mass balance, respectively.

For DON in BSF, the average mass balance ranged from 39 to 55% in the individual treatments and from 55 to 80% in the mixture treatments (Figure 3). While the majority of DON was measured as parent compound (original mycotoxin the metabolite originates from) in the RM (spiked feed), up to an average of 2% was measured in RM (gut clean). For the LMW, the mass balance was similar for the individual treatments and the mixture and ranged from 80 to 96%. In both the BSF and the LMW, the three DON metabolites were below the LOQ in the larvae as well as the two residual materials and hence were not included in the mass balance. Setting measurements for the metabolites below LOQ to half the LOQ contributed to less than 1% of the overall mass balance for DON.

For ZEN in the LMW, the mass balance shows that between 88% and 117% of the mass can be explained; 7–10% of this mass originated from the presence of the two metabolites in the RM (spiked feed). In the BSF, between 101% and 141% of the mass was accounted for, with the metabolites contributing to a larger extent of the overall mass balance than for the LMW. Metabolites in the RM (spiked feed) contributed to an average maximum percentage of 86% of the overall mass balance for ZEN in the BSF.

OTA was found in the BSF larvae (except for the L1 and M1 treatments) and both residual materials in all BSF treatments, in total accounting for 41–62% of the mass as parent compound (no OTA metabolites were included in this study). The mass in the larvae and in the RM (gut clean) account for less than 1% of the overall mass balance in each treatment. In the LMW, OTA was absent in the larvae (single and mix treatments) but present in the RM (spiked feed), accounting 97–126% of the spiked feed and the RM (gut clean) at less than 1% of the overall mass.

## 3. Discussion

This study investigated the potential accumulation and/or excretion of the mycotoxins AfB1, DON, ZEN. and OTA in insect larvae of the lesser mealworm (*Alphitobius diaperinus,* LMW) and the black soldier fly (*Hermetia illucens*, BSF) reared on feed spiked with single mycotoxins and on feed spiked with a mixture of the four mycotoxins. To our knowledge, this is the first study investigating the potential accumulation/excretion of LMW for ZEN and OTA. Additionally, it is the first controlled study investigating the fate of a mixture of mycotoxins—AfB1, DON, ZEN, and OTA—relative to the single mycotoxins when feeding these insect larvae with contaminated feed. 

### 3.1. Insect Performance

The survival rate of the BSF and LMW reared on single mycotoxins and the mixture of all mycotoxins after 10 and 14 days, respectively, was comparable to the survival rate of the larvae reared on “mycotoxin-free” feed (control), indicating that at the exposure levels used in this study, the larvae of both species could tolerate high levels of mycotoxins in their feed without impacting their survival rate. This is true also for their growth rate such that, generally, the larvae reared on mycotoxin spiked feed had a similar growth rate to the larvae reared on control feed, further indicating the tolerance of these larvae to being reared on high concentrations of AfB1, DON, ZEN, and OTA in their feed comparable to other studies [12]. At the end of the experiment, the average live weight of the BSF (14 days old) ranged from 172 to 191 mg, which is comparable to previous studies [10,13]. Our study showed that accumulation/excretion of the mycotoxins by both insect larvae was independent of the presence of any other of the mycotoxins included in this study, especially exposed to all four mycotoxins, approximately 25 times the EC maximum limit. This result is particularly important since crops contaminated with mycotoxins will generally contain multiple mycotoxins contaminated to different degrees.

### 3.2. Aflatoxin B_1_

In the study of Bosch et al. [10], the accumulation/excretion of AfB1 in BSF and yellow mealworm (*Tenebrio molitor*, YMW) larvae was investigated using substrate spiked to six different levels of AfB1 ranging from 0.01 to 0.5 mg/kg dry feed. Their highest level was similar to L3 in the current study. Concentrations of AfB1 in larvae of BSF were below the limit of detection of 0.1 µg/kg, while AfB1 was detected in YMW larvae only when reared on feed containing 0.023 mg/kg AfB1 or more [10]. In our study, AfB1 was below the quantification limit of 1 µg/kg dw in both the BSF and LMW larvae, which confirms the results for BSF obtained in previous studies [10].

The mass balance for AfB1 resulted in less than 20% of the parent compound spiked in the feed could be accounted for in the BSF treatments. This result also confirms results obtained in previous studies [10]. While in previous work [10] only the metabolite AfM1 was analysed for, in our study, three additional aflatoxin metabolites, namely aflatoxicol, aflatoxin P_1_, and aflatoxin Q_1_, were included. However, the concentrations of these additional metabolites still could not explain the mass unaccounted for. While previously similar mass balance results were obtained for the YMW [10], in this study, the mass balance for the LMW ranged between 56% and 80%. This indicates that the LMW has a lower capacity to metabolize AfB1 than the BSF and YMW. Various insect species have previously been found to metabolize AfB1 into its more toxic (AFB0) or less toxic (AfM1, Q1 and P1) metabolites [14]. More recent research has focused on the function of cytochrome P450 monooxygenases in the detoxification of AfB1 in, inter alia, honeybees (*Apis mellifera*) [15], orange navelworm (*Amyelois transitella*) [14], and corn earworm (*Helicoverpa zea*) [16]. Our study, and previous research indicates that the extent to which such insects metabolise AfB1 appears to vary between insect species, and additional toxicological research is recommended beyond the additional metabolites included in this study.

### 3.3. Deoxynivalenol

The fate of DON in YMW larvae was also recently investigated by Van Broekhoven et al. [9], where YMW larvae were reared on both naturally contaminated wheat flour (concentration of 4.9 mg DON/kg) and on wheat flour spiked with DON (concentration of 8 mg DON/kg). After harvesting the 14-day-old larvae, concentrations of the parent compound DON and the DON metabolites 15- and 3-acetyl-DON and DON-3-glucoside were all below the limit of detection of 100 μg/kg in the larvae. In our study, similar results for the parent compound and metabolites were obtained for the LMW. However, due to matrix interference, the LOQ in our study was several times higher for the metabolites. Very low levels of DON were detected in the larvae of the BSF at concentrations much lower than was available in the feed. When naturally contaminated flour was used for rearing, about 14% of the amount of DON in the substrate was excreted by the larvae, compared with 41% excretion in case of the spiked wheat flour [9]. The remaining DON fraction could not be accounted for by the levels of the DON and the three metabolites in the larvae and the excreta. The authors of that study could not explain the differences in DON excretion of the larvae grown on the naturally contaminated and spiked wheat flour and suggested it could possibly be due to other fungal metabolites in the naturally contaminated wheat flour. In our study, 55–80% of DON was accounted for in the BSF reared on the mixtures, and 39–55% when the BSF were reared solely on DON contaminated feed. Furthermore, between 80% and 96% of the DON was accounted for in the LMW treatments. A clear difference can thus be observed between the different insect species, in their potential to metabolize DON. It remains unclear what the unaccounted mass for DON may be.

In the study by Purschke et al. [12], after 10 days of exposure to AfB1, DON, ZEN, and OTA at concentrations also used in this study, all mycotoxins were below the respective limit of detection in the larvae of the BSF as well as the residual material for aflatoxin B_2_, aflatoxin G_2_ and OTA. Concentrations of AfB1 and ZEN in the residual material were comparable to levels in the initial substrate. Additionally, DON concentrations in the residual material were significantly higher than in the starting material; almost double the amount originally present. All these trends between feed and residual material are comparable to results obtained in this study for the larvae of the BSF. In the study by Purschke et al. [12], the authors speculated that the increase in DON concentration from feed to the final residual material could be due to mycotoxin derivatives bound to the carbohydrate or protein matrix (claimed to be undetectable by conventional techniques) reverting back to the original chemical structure during the trial. However, in their study, no mass balance was performed. In this study, we showed that while the concentration of DON in the RM (spiked feed) was greater than the feed, the overall mass balance was less than 100% indicating that DON was in fact metabolized by the BFS larvae.

### 3.4. Zearalenone and Ochratoxin A

In this study, more than 88% was accounted for in both ZEN and OTA treatments for the LMW, indicating that the larvae of the LMW excreted the majority of ZEN and OTA taken up from their feed, and metabolize these mycotoxins only to a small extent. Van Broekhoven et al. [17] assessed the tolerance and accumulation of ZEN, OTA and T-2 toxin in Tenebrionid beetle larvae (*Tenebrio molitor*, *Zophobas atratus* and *Alphitobius diaperinus*). They found that although these mycotoxins were initially retained in the larvae, the concentrations dropped rapidly when the larvae fasted before harvesting—which is considered a standard practice in commercial insect rearing [5]. In the case of YMW, the ZEN and OTA levels dropped from 42.0 μg/kg and 2.5 μg/kg dry weight, respectively, to undetectable levels after 24 h of fasting [17].

More than 50% of the mass balance of ZEN in the BSF treatments was made up of the two metabolites, α- and β-zearalenol. Based on the uterotrophic activity assessed in rodents, α-zearalenol is more toxic than its parent compound ZEN, while β-zearalenol is less toxic than its parent compound [18]. The percentage of the metabolites in the ZEN mass balance between the mixture and the single mycotoxin is comparable, which indicates that the insect larvae metabolisms are independent of the presence of other mycotoxins. While this is true for all the mycotoxins included in this study, this is most clear for ZEN, given the higher percentage of metabolites in the residual material. Roughly half of the OTA was accounted for in the BSF, which is in strong contrast with the LMW, where more than 97% of the OTA was accounted for. OTA is the main mycotoxin in the group of ochratoxin mycotoxins and the only one of major carcinogenic significance. OTA B, the decholoroninated derivate of OTA, is not toxic [19]. OTA metabolism by insects has been investigated but could not be confirmed [14,15], but several reports of OTA biodegradation by bacteria and yeast have been published [20].

### 3.5. Regulations

In this study, the substrates were spiked to levels relative to EC maximum or guidance limits, so as to study the potential accumulation of the contaminants in the larvae, relative to the (safe) limits applied to ingredients used for feed and food products. In addition to the particular EC limit, we also intended to spike to a 10- and 25-fold increase of this limit. Results of this study clearly showed that in all cases, the two larvae species complied with the EC limits. Since both insect species either excreted or metabolized the mycotoxin present in their feed, even with the mixtures treatments with an average of 8- and 20-fold increase of the limit, the safe limits for mycotoxins in feed intended to be used as substrate for growing insects may possibly be higher than the current EC limits for feed for production animals.

### 3.6. Further Research

This study investigated the potential accumulation of mycotoxins in two of the insect types that are currently considered for feed and food use in Europe. In addition to the four mycotoxins, some well-known metabolites were analyzed as well. Results of the mass balance for **AfB1s** and DON in BSF indicate this insect species may form other metabolites, such as de-epoxynivalenol (DOM)—a metabolite of DON which can be produced by bacteria in the gut—or conjungated forms of mycotoxins. Further analytical and toxicological research is therefore needed to obtain insights into mycotoxin metabolism of BSF and LMW and to fully understand the safe limits of mycotoxins in insect feed and thus the safety of the insects.

## 4. Materials and Methods

**Figure 4:**
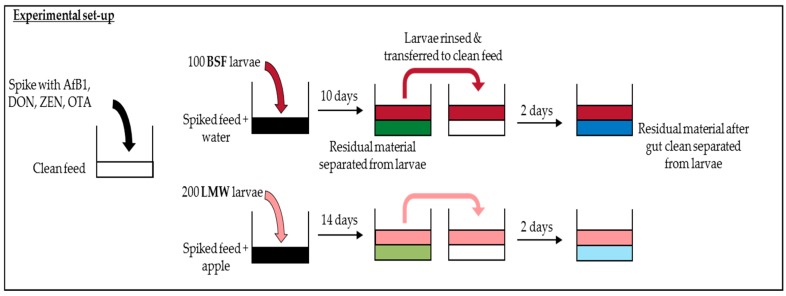
Grpahical illustration of the experiment set-up.

An experiment was conducted in which two insect species were exposed to individual mycotoxins (AfB1, DON, OTA, ZEN) as well as to a mixture of the four mycotoxins via feed contaminated at different concentrations. Insects were grown on the contaminated feed. At the end of the exposure period the insects were separated from the residual material (RM)—which consisted of remaining feed, frass, and excuviae—and analyzed collectively (hereafter referred to as RM (spiked feed)). Post exposure, the insects were given clean feed for two days in order to clean their gut of contaminated feed, after which the insects were again separated from the residual material (hereafter referred to as RM (gut clean)). Gut cleaning for two days was included since we aimed to investigate potential accumulation of the mycotoxin in the larvae, not in their gut (containing spiked substrate given to the larvae). Insect starvation is common practice in rearing insects for feed and food consumption. All samples (larvae and both residual materials) were analysed for the four mycotoxins and their metabolites. The metabolites that were analyzed are those for which reference material is available, and thus can be quantified. The experiment is illustrated in Figure 4 and described in detail below.

### 4.1. Feed Preparation

Standard wheat-based feed (layer meal based feed) used for commercial rearing the LMW and BSF larvae was supplied by Proti-Farm BV, Ermelo, the Netherlands. Individual batches of feed were spiked to three different levels of AfB1 (*Aspergillus flavus,* 99.6% purity, Sigma-Aldrich, Saint Louis, MO, USA), DON (99.5% purity, Sigma-Aldrich, Saint Louis, MO, USA), ZEN (fungus, 99.7% purity, Sigma-Aldrich, Saint Louis, MO, USA), and OTA (99.3% purity, Sigma-Aldrich, Saint Louis, MO, USA) as listed in Table 1. Spiking was performed externally by Ducares B.V., Utrecht, The Netherlands. First, 0.25 mg AfB1, 62.5 mg DON, 6.25 mg ZEN, and 1.25 mg OTA were individually dissolved in a minimum amount of chloroform, ethanol, methanol and chloroform, respectively, and then diluted in 10 mL methanol. These solutions were then sprayed on 500 g of feed and vaporized to dryness in a flow-cabinet by air. After a day, the feed was mixed. This resulting feed was contaminated at the concentration of L3 (Table 1). From L3, the L2 feed samples were made by diluting with clean feed, and similarly, L1 was made from L2. For the feed spiked with the mixture of mycotoxins, the same amounts of mycotoxins were dissolved in the same solvent mentioned above and then combined and diluted to 10 mL of methanol. This solution containing a mixture of mycotoxins was sprayed on the feed, and dilutions of feed were made as described above resulting in feed contaminated at levels M3, M2, and M1 (Table 1).

After spiking, thorough mixing of the feed was performed by placing the containers with the spiked feed on a shaker for one hour. In order to verify the homogeneity of the feed, homogeneity tests were performed on the feed spiked with the mixture of mycotoxins at the lowest concentrations (M1). Ten individual samples of the spiked feed of ML1 were analyzed according to the method described in Section 4.3.

### 4.2. Experimental Set-Up

The experiment was executed with two insect species—BSF and LMW—and 15 treatments per insect species. The 15 treatments included three different concentrations for each of AfB1, DON, ZEN, and OTA individually and three concentrations levels for a mixture of these four mycotoxins. Each treatment was performed in triplicate. Insects were reared on two separate control treatments, specifically (i) un-spiked (control) feed and (ii) feed spiked with solvent—methanol (control solvent). The intended spiking concentration of treatment L1 for AfB1 was 0.02 mg/kg (at moisture content of 12%), corresponding to the (European) maximum allowed AfB1 concentration in feed materials as stated in Directive 2002/32/EC (consolidated version 27 February 2015). The treatments L1 levels for DON, ZEN and OTA were aimed to spike to a concentration of 0.1, 0.5, and 5 mg/kg, respectively, according to the respective mycotoxin maximum guidance value for complete feeding stuffs, as stated in European Commission Recommendation 2006/576/EC. Treatments L2 and L3 were aimed to be spiked to concentration of 10 times and 25 times L1, respectively (Table 1). Three more mixture treatments were included, namely M1, M2, and M3, which were spiked with all four mycotoxins to their respective aimed concentrations in levels 1–3.

Per replicate, 100 one-week-old BSF larvae were placed in a plastic petri dish (9 × 4 cm) with ventilated lids. Each treatment contained 20 g of the respective spiked feed and 30 mL of water (water source). After 10 days, the larvae were separated from the residual material (remaining feed, frass, and exuviae), rinsed with tap water, dried, and transferred to another container with 10.0 g of unspiked feed and 10 mL of water. After two days, the larvae were again separated from the residual material and cleaned and then harvested.

Per replicate, 200 two-week-old LMW larvae were placed in a plastic container (5 × 5 × 3 cm) with 20.0 g of the respective treatment feed and a piece of apple as a water source. Weights of the larvae were determined before they were put in the container. An additional piece of apple was added approximately every three days to ensure continuous access to water. After 14 days, the larvae were separated from the residual material, rinsed with tap water, dried and transferred to another container with 10.0 g of control feed, and a piece of apple. After two days, the larvae were again separated from the residual material, cleaned and then harvested.

After each step, the weights of the larvae were determined after sieving, washing with water, and drying the larvae. Also, the numbers of larvae were counted with a counting machine. Weights of the residual material were determined after each of the two steps.

Both insect species were reared under standard growing conditions in a climate chamber at the respective company: the LMW larvae at Proti-farm BV at 28–29 °C and 50–60% relative humidity, and the BSF larvae at Koppert B.V. at 26 °C, 80% relative humidity, LD 12:12. Insects and residual material samples were freeze-dried and subsequently stored at −20 °C until further analysis.

### 4.3. Sample Preparation and Mycotoxin Analyses

#### 4.3.1. Standard and Chemicals

Mycotoxin standards used were obtained from Biopure (Oostvoorne, the Netherlands), Sigma Aldrich (Zwijndrecht, The Netherlands), Enzo Life Sciences (Brussels, Belgium) and TRC (Toronto, ON, Canada). Eight mycotoxin metabolites were measured in the larvae and in both residual materials; namely aflatoxicol (AfOL), aflatoxin P_1_ (AfP1), aflatoxin Q_1_ (AfQ1), aflatoxin M_1_, (AfM1), 3-acetyl DON (3-ac-DON), 15-acetyl DON (15-ac-DON), DON-3-glucoside (DON-3-Glc), α-zearalenol (α-ZEN), and β-zearalenol (β-ZEN).

#### 4.3.2. Extraction and Chemical Analyses

A validated and accredited LC-MS/MS-based method for the analysis of mycotoxins in feed and food materials was used for quantification of AfB1, DON, ZEN, and OTA. The scope of this method was extended in order to quantify also the mycotoxins and their metabolites in larvae and excreta of BSF and LMW. In the MS/MS method, two MRM transitions were included for each mycotoxin, details on this and additional MS/MS settings can be found in Tables S4 and S5 of the Supplementary Materials. Each mycotoxin was identified by its retention time and the peak area ratio between two transitions: the quantifier and the qualifier (Tables S4 and S5). Quantification was performed by bracketed calibration (an interval of not more than 10 injections) on the peak area of the quantifier (qn) of matrix-matched calibration solutions (MMS). Concentrations of AfB1, DON, ZEN, and OTA were corrected for matrix effects with the use of their respective ^13^C-isotope labeled standards.

The limit of quantification (LOQ) was defined as the lowest calibrated level which complied with the required QC parameters as mentioned in SANTE/11945/2015 and the limit of detection (LOD) was derived from the signal-to-noise (s/n) ratio of the lowest matrix-matched standard solutions and varied between mycotoxins. Mycotoxin-specific LOQs can be found in Table S2. The performance of the analytical method was tested by performing repeated analyses of a control sample (*n* = 6). Average recoveries and within lab reproducibility (RSDwR) are presented in Table S6.

From each treatment 2.5 g was weighed in a 50 mL plastic tube and 7.5 mL of water was added. After 15 min, 10 mL of extraction solvent (acetonitrile/acetic acid 99:1 (*v*/*v*)) and 25 µL of ^13^C-caffeine internal standard (IS) were added (in case less material was available 1.0 g of material was taken and the extraction volume and IS amount were adjusted accordingly). The tubes were shaken manually to loosen the solids on the bottom of the tube and put in a horizontal shaking device (Edmund Bühler SM30 Control, Hechingen, Germany) (200 shakes min^−1^) for 0.5 h. Anhydrous magnesium sulphate (4 g) was added, and the mixture was vortexed for 1 min. Subsequently, the samples were centrifuged for 10 min at 3500 rpm, and 200 µL of sample extract was transferred to polypropylene vials (Whatman syringeless filter device). To each vial 180 µL of water was added, and 20 µL of a solution containing ^13^C isotope labels of AfB1, DON, ZEN, and OTA. The vials were capped and vortexed for approximately 3 s. After 30 min in the refrigerator (4 °C) the vials were closed with a pressing device (Six Position Compression; Whatman,‘s-Hertogenbosch, The Netherlands) and stored at 4 °C until LC-MS/MS analysis.

The LC-MS/MS system consisted of an injection and pump system from Shimadzu (DGU-20A3 degasser; SIL-20AC XR autosampler; LC-20AD XR pump and CTO-20AC column oven; Shimadzu’s, Hertogenbosch, The Netherlands) and an AB Sciex QTRAP 5500 MS equipped with an ESI source operated in positive and negative mode (AB Sciex, Nieuwerkerk a/d IJssel, The Netherlands). Samples were kept at 10 °C, and a volume of 5 µL was injected into the LC-MS/MS system. For LC separation, a 100 mm × 2.1 mm ID, 3 µm Restek Ultra Aqueous C18 column (Interscience, Breda, The Netherlands) was used. The LC mobile phases for the analyses in positive ionization mode were water (A) and methanol:water 95:5 (B) both containing 1 mM ammonium formate and 1% formic acid, while for negative ionization mode the mobile phases consisted of water (A) and methanol:water 95:5 (B) both containing 5 mM ammonium acetate and 0.1% acetic acid. The LC eluent gradient for both the negative and positive ionisation modes were: 1 min isocratic at 100% A, then a linear gradient to 50% B in 2 min followed by a linear gradient to 100% B in 8 min. For complete elution of all matrix co-extractants from the column, the final composition at 100% B was kept for 2 min. In half a minute, the initial conditions were restored and then equilibrated for 5 min before the next injection. The LC flow rate was 400 µL/min. The temperature of the column oven was 35 °C. Electrospray ionization conditions were set as follows: temperature 400 °C, spray voltage 4.0 kV/−4.0 kV, GS1 50 arbitrary units, GS2 50 arbitrary units.

### 4.4. Data Analysis

The larvae survival rate and the larval live weight at harvest were quantified in order to determine the effect of the mycotoxin exposure on larval development (performance). One-way analysis of variance (ANOVA), followed by Tukeys HSD posthoc test, were used to determine whether differences in the means of the individual treatments were significant, at a significance level of 0.05 (IBM SPSS Statistics 23). T-tests were used to determine whether differences in the mycotoxin concentrations between the feed and RM (spiked feed) were significant at a significance level of 0.05.

For all treatments other than the control treatments, the mass balance on the respective mycotoxin was determined. The mass in the larvae and in both residual material samples was quantified. Mycotoxins and metabolites concentrations that were below the limit of quantification (LOQ) were set to zero for the mass balance calculations. The sum of the mass in the larvae and in both residual materials was then divided by the mass of the respective mycotoxin in the feed, and the percentage of the detected mass was determined.

## Figures and Tables

**Table 1:** Intended and average (relative standard deviation (%) (*n* = 3)) analysed spiking concentrations of mycotoxins in feed in mg/kg wet weight (ww).

	Mycotoxin Concentration (mg/kg ww)								
	Level 1			Level 2			Level 3		
Mycotoxin	Intended	Analysed (Single) L1	Analysed (Mix) M1	Intended	Analysed (Single) L2	Analysed (Mix) M2	Intended	Analysed (Single) L3	Analysed (Mix) M3
AfB1	0.02	0.008 (16)	0.018 (2.7)	0.2	0.07 (13)	0.18 (7.4)	0.5	0.39 (12.0)	0.43 (1.8)
DON	5	3.9 (6.0)	4.1 (2.4)	50	38 (3.0)	41 (9.0)	125	112 (1.8)	100 (1.3)
ZEN	0.5	0.28 (4.9)	0.4 (3.1)	5	2.5 (2.5)	3.8 (8.9)	12.5	13 (5.1)	9.4 (2.9)
OTA	0.1	0.17 (4.0)	0.08 (4.1)	1	1.7 (7.0)	0.8(6.5)	2.5	1.3 (11.0)	2 (4.3)

**Table 2:** Concentration in mg/kg dry matter (dw) (± standard deviation) of detectable aflatoxin B_1_ metabolites aflatoxicol and aflatoxin M_1_ and detectable zearalenone metabolites α- and β-zearalenol in the larvae, RM (spiked feed) and RM (gut clean) of individual and mixture mycotoxin treatments of the black soldier fly (BSF) and the lesser mealworm (LMW) ^a^. The aflatoxin B_1_ metabolites aflatoxin P_1_ and aflatoxin Q_1_ were below their respective LOQ in all treatments.

		Aflatoxicol (Metabolite 1)			Aflatoxin M_1_ (Metabolite 2)		
		Larvae	Residual Material (Spiked Feed)	Residual Material (Gut Clean)	Larvae	Residual Material (Spiked Feed)	Residual Material (Gut Clean)
BSF	L1	<0.001 ^b^	<0.005	<0.005	<0.001	<0.001	<0.001
	L2	<0.001	<0.005	<0.005	<0.001	<0.001	<0.001
	L3	<0.001	<0.005	<0.005	<0.001	<0.001	<0.001
	M1	<0.001	<0.005	<0.005	<0.001	<0.001	<0.001
	M2	<0.001	<0.005	<0.005	<0.001	<0.001	<0.001
	M3	<0.001	0.067 ± 0.002	<0.005	<0.001	<0.001	<0.001
LMW	L1	<0.001	<0.001	<0.001	<0.001	<0.001	<0.001
	L2	<0.001	<0.001	<0.001	<0.001	0.0020 ± 0.0002	<0.001
	L3	<0.001	0.0015 ± 0.000	<0.001	<0.001	0.011 ± 0.0001	<0.001
	M1	<0.001	<0.001	<0.001	<0.001	<0.001	<0.001
	M2	<0.001	<0.001	<0.001	<0.001	0.0055 ± 0.001	<0.001
	M3	<0.001	0.0013 ± 0.0002	<0.001	<0.001	0.011 ± 0.001	<0.001
		**α-Zearalenol (Metabolite 1)**			**β-Zearalenol (Metabolite 2)**		
BSF	L1	<0.005	0.64 ± 0.026	<0.02	<0.005	0.18 ± 0.010	<0.01
	L2	0.005 ± 0.002	7.2 ± 0.31	0.14 ± 0.04	<0.005	2.3 ± 0.12	0.042 ± 0.02
	L3	0.025 ± 0.004	37.3 ± 8.1	0.60 ± 0.18	0.007 ± 0.001	11.2 ± 1.32	0.18 ± 0.06
	M1	<0.005	0.74 ± 0.11	0.022 ± 0.002	<0.005	0.20 ± 0.026	<0.02
	M2	0.011 ± 0.004	12.7 ± 2.9	0.21 ± 0.012	<0.005	3.6 ± 0.85	0.071 ± 0.051
	M3	0.029 ± 0.005	28.3 ± 1.5	0.63 ± 0.078	0.0067 ± 0.001	8.6 ± 1.02	0.13 ± 0.032
LMW	L1	<0.005	0.023 ± 0.002	<0.02	<0.005	<0.01	<0.01
	L2	<0.005	0.203 ± 0.012	<0.02	<0.005	0.102 ± 0.007	<0.01
	L3	<0.005	0.82 ± 0.071	<0.02	<0.005	0.32 ± 0.026	<0.01
	M1	<0.005	0.030 ± 0.002	<0.02	<0.005	0.011 ± 0.001	<0.01
	M2	<0.005	0.35 ± 0.040	<0.02	<0.005	0.14 ± 0.012	<0.01
	M3	<0.005	0.74 ± 0.012	<0.02	<0.005	0.27 ± 0.015	<0.01

^a^ Other AfB1, ZEN, OTA, and DON metabolites were not detected in any of the matrices. ^b^ Concentration was below the LOQ, therefore the LOQ is provided in the table.

## Supplementary Materials

The following are available online at http://www.mdpi.com/2072-6651/10/2/91/s1, Table S1: Survival rate (%), live weights (mg) and dry matter content (%), all including the standard deviation (*n* = 3) of the black soldier fly and the lesser mealworm larvae at the different treatments, Table S2: Concentrations (±standard deviation (*n* = 3) of mycotoxins AfB1, DON, ZEN and OTA in larvae, residual material (spiked feed) and residual material (clean gut), Table S3: Average mass balance percentages (±standard deviation) *n* = 3) include metabolites measured above the limit of quantification, Table S4: Instrumental MS/MS parameters of mycotoxins analysed in positive ionisation mode, Table S5: Instrumental MS/MS parameters of mycotoxins analysed in negative ionisation mode, Table S6: Average recovery and within-lab reproducibility of mycotoxins in repeated analyses of a control sample (*n* = 6).
