# Supplementary material for: Tolerance and Excretion of the Mycotoxins Aflatoxin B1, Zearalenone, Deoxynivalenol, and Ochratoxin A by Alphitobius diaperinus and Hermetia illucens from Contaminated Substrates
Source: Toxins (Basel). 2018 Feb 24;10(2):91. doi: 10.3390/toxins10020091 (PMC5848191; doi:10.3390/toxins10020091)
Supplement: Supplementary file 1 [file toxins-10-00091-s001.pdf]

Supplementary Materials: Tolerance and excretion of the mycotoxins aflatoxin B1, zearalenone, deoxynivalenol and ochratoxin A by *Alphitobius diaperinus* and *Hermetia illucens* from contaminated substrates

Louise Camenzuli, Ruud Van Dam, Rob Andriessen, Jeroen Van Schelt, and H.J. (Ine) Van der Fels-Klerx

Table S1. Survival rate (%), live weights (mg) and dry matter content (%), all including the standard deviation (n=3) of the black soldier fly and the lesser mealworm larvae at the different treatments.

		Black soldier fly larvae			Lesser mealworm larvae		
		Survival rate (%)	Live weight per larva (mg)	Dry matter content (%)	Survival rate (%)	Live weight per larva (mg)	Dry matter content (%)
Control		98 ± 1	180.6 ± 0.9	40.3 ± 1.0	74 ± 2	19.6 ± 0.8	33.2 ± 0.2
Control + Solvent		99 ± 0	178.8 ± 1.4	39.8 ± 0.0	82 ± 2	18.4 ± 0.5	33.0 ± 0.1
AfB1	Level 1	100 ± 1	172.3 ± 1.1	41.2 ± 0.2	81 ± 4	18.2 ± 1.4	33.4 ± 0.6
	Level 2	98 ± 2	185.4 ± 4.4	39.4 ± 0.2	83 ± 4	17.0 ± 0.9	32.6 ± 0.3
	Level 3	98 ± 1	185.8 ± 6.3	38.9 ± 0.4	79 ± 4	18.9 ± 1.2	32.9 ± 0.3
DON	Level 1	98 ± 2	188.2 ± 4.4	38.2 ± 0.9	75 ± 8	16.7 ± 1.1	32.4 ± 0.5
	Level 2	99 ± 1	184.5 ± 3.0	38.3 ± 0.1	77 ± 2	17.3 ± 1.5	33.2 ± 0.1
	Level 3	98 ± 2	186.8 ± 4.3	38.2 ± 0.3	81 ± 5	17.2 ± 0.2	33.4 ± 0.2
ZEN	Level 1	98 ± 2	189.9 ± 6.6	38.7 ± 0.7	78 ± 2	18.8 ± 1.6	33.4 ± 0.9
	Level 2	99 ± 1	185.6 ± 4.1	40.5 ± 1.9	71 ± 5	16.8 ± 1.3	33.0 ± 0.1
	Level 3	96 ± 2	184.8 ± 2.7	40.0 ± 1.6	80 ± 2	17.4 ± 0.7	33.1 ± 0.1
OTA	Level 1	98 ± 2	188.7 ± 1.2	37.6 ± 0.3	78 ± 4	17.3 ± 0.3	33.3 ± 0.1
	Level 2	98 ± 1	181.0 ± 5.5	39.0 ± 0.3	79 ± 4	19.5 ± 0.3	33.8 ± 0.5
	Level 3	98 ± 1	190.9 ± 5.4	39.7 ± 1.3	77 ± 3	16.4 ± 0.8	32.9 ± 0.5
Mixture	Level 1	98 ± 2	187.4 ± 3.7	39.1 ± 0.7	79 ± 0	16.9 ± 1.3	32.8 ± 0.3
	Level 2	94 ± 7	188.9 ± 3.1	38.2 ± 0.1	75 ± 7	19.9 ± 0.5	34.5 ± 0.3
	Level 3	98 ± 2	191.2 ± 4.2	38.7 ± 1.0	71 ± 8	18.8 ± 0.2	34.1 ± 0.3

Table S2. Concentrations (± standard deviation (n=3) of mycotoxins AfB1, DON, ZEN and OTA in larvae, residual material (spiked feed) and residual material (gut clean) of the black soldier fly (BSF) and lesser mealworm (LMW). Note respective units above each section.

		Black solider fly larvae			Lesser mealworm larvae		
		Larvae	Residual material (spiked feed)	Residual material (gut clean)	Larvae	Residual material (spiked feed)	Residual material (gut clean)
		µg/kg$_{dw}$	µg/kg$_{dw}$	µg/kg$_{dw}$	µg/kg$_{dw}$	µg/kg$_{dw}$	µg/kg$_{dw}$
Control		<LOQ	<LOQ	<LOQ	<LOQ	<LOQ	<LOQ
Control + solvent		<LOQ	<LOQ	<LOQ	<LOQ	<LOQ	<LOQ
LOQ		1	1	1	1	1	1
AfB1	L1	<1	5.5±0.5	<1	<1	5.7±0.4	<1
	L2	<1	62.0±3.6	1.8±0.1	<1	61.7±1.5	<1
	L3	<1	303.3±15.3	5.3±1.0	<1	326.7±11.5	<1
	M1	<1	13.7±1.2	<1	<1	17.7±1.2	<1
	M2	<1	156.7±20.8	1.8±0.1	<1	153.3±5.8	<1
	M3	<1	353.3±25.2	4.6±0.1	<1	413.3±11.5	<1
		µg/kg$_{dw}$	mg/kg$_{dw}$	mg/kg$_{dw}$	µg/kg$_{dw}$	mg/kg$_{dw}$	µg/kg$_{dw}$
LOQ		80	0.2	0.2	200	0.2	200
DON	L1	<80	7.7±2.9	0.3±0.07	<200	4.3±0.2	<200
	L2	129.3±31.0	86.3±25.7	2.5±0.5	<200	44.7±2.1	<200
	L3	256.7±15.3	316.7±60.2	5.5±1.2	<200	110.0±0.0	263.3±5.8
	M1	<80	15.7±0.6	0.2±0.04	<200	4.7±0.2	<200
	M2	109.5±29.0	150.0±17.3	1.8±0.1	<200	47.3±2.1	<200
	M3	176.7±11.5	296.7±11.6	4.2±0.7	<200	116.7±5.8	<200
		µg/kg$_{dw}$	mg/kg$_{dw}$	µg/kg$_{dw}$	µg/kg$_{dw}$	mg/kg$_{dw}$	µg/kg$_{dw}$
LOQ		20	0.02	20	20	0.02	20

ZEN	L1	<20	0.7±0.04	<20	<20	0.28±0.01	<20
	L2	<20	6.6±0.4	183.3±35.1	<20	3.0±0.06	<20
	L3	27.5±6.4	35.3±3.2	690.0±192.9	<20	13.7±1.2	<20
	M1	<20	0.99±0.1	<20	<20	0.49±0.03	<20
	M2	<20	10.2±0.8	78.7±5.8	<20	4.3±0.06	<20
	M3	<20	22.7±2.1	226.7±106.9	<20	12.7±0.6	<20
		µg/kg$_{dw}$	mg/kg$_{dw}$	µg/kg$_{dw}$	µg/kg$_{dw}$	mg/kg$_{dw}$	µg/kg$_{dw}$
LOQ		1	0.002	2	1	0.002	2
OTA	L1	<1	0.4±0.03	5.1±0.7	<1	0.21±0.01	<2
	L2	2.6±0.6	5.1±0.6	45.0±8.7	<1	2.4±0.2	<2
	L3	2.2±0.9	3.5±0.3	26.3±2.5	<1	1.9±0.1	<2
	M1	<1	0.2±0.02	<2	<1	0.12±0.01	<2
	M2	1.8±0.8	2.5±0.3	27.3±4.2	<1	1.1±0.06	<2
	M3	3.9±0.9	5.0±0.5	69.0±16.4	<1	3.1±0.1	2.4±0.4

Table S3: Average mass balance percentages (± standard deviation) n=3) include metabolites measured above the limit of quantification. See Table 2 for detected Aflatoxin B1 (Metabolite 1= Aflatoxicol; metabolite 2 = Aflatoxin M1) and zearalenone (Metabolite 1= α-zearalenol; metabolite 2 = β-zearalenol) metabolites.

Average and SD for mass balance results

LMW		% parent (larvae)	% metabolite 1 (larvae)	% metabolite 2 (larvae)	% parent (RM spiked feed)	% metabolite 1 (RM spiked feed)	% metabolite 2 (RM spiked feed)	% parent (RM gut clean)	% metabolite 1 (RM gut clean)	% metabolite 2 (RM gut clean)
AfB1	L1	0	0	0	56±4	0	0	0	0	0
	L2	0	0	0	77±2	0	2±0.3	0	0	0
	L3	0	0	0	67±2	0.3±0.01	2±0.2	0	0	0
	M1	0	0	0	79±5	0	0	0	0	0
	M2	0	0	0	68±3	0	3±0.5	0	0	0
	M3	0	0	0	78±3	0.3±0.04	2±0.1	0	0	0
DON	L1	0	0	0	90±3	0	0	0	0	0
	L2	0	0	0	96±4	0	0	0	0	0
	L3	0	0	0	80±1	0	0	0.1±0	0	0
	M1	0	0	0	92±3	0	0	0	0	0
	M2	0	0	0	91±3	0	0	0	0	0
	M3	0	0	0	94±5	0	0	0	0	0
ZEN	L1	0	0	0	81±1	7±0.6	0	0	0	0
	L2	0	0	0	99±2	7±0.4	3±0.3	0	0	0
	L3	0	0	0	84±6	5±0.5	2±0.2	0	0	0
	M1	0	0	0	98±6	6±0.5	1±1	0	0	0
	M2	0	0	0	90±2	7±0.9	3±0.3	0	0	0
	M3	0	0	0	109±4	6±0.1	2±0.1	0	0	0
OTA	L1	0	0	0	97±4	0	0	0	0	0
	L2	0	0	0	111±9	0	0	0	0	0
	L3	0	0	0	115±5	0	0	0	0	0
	M1	0	0	0	116±5	0	0	0	0	0
	M2	0	0	0	111±6	0	0	0.05±0.08	0	0
	M3	0	0	0	126±3	0	0	0.05±0.01	0	0

BSF		% parent (larvae)	% metabolite 1 (larvae)	% metabolite 2 (larvae)	% parent (RM spiked feed)	% metabolite 1 (RM spiked feed)	% metabolite 2 (RM spiked feed)	% parent (RM gut clean)	% metabolite 1 (RM gut clean)	% metabolite 2 (RM gut clean)
AfB1	L1	0	0	0	11±0.9	0	0	0	0	0
	L2	0	0	0	17±1	0	0	0.5±0.4	0	0
	L3	0	0	0	15±1	0.5±0.1	0	0.4±0.05	0	0
	M1	0	0	0	16±1	0	0	0	0	0
	M2	0	0	0	17±2	0	0	0.3±0.05	0	0
	M3	0	0	0	15±3	0.2±0.2	0	0.3±0.02	0	0
DON	L1	0	0	0	37±15	0	0	2±0.5	0	0
	L2	0.2±0.03	0	0	43±12	0	0	2±0.3	0	0
	L3	0.08±0.00	0	0	54±15	0	0	1±0.2	0	0
	M1	0	0	0	79±4	0	0	1±0.2	0	0
	M2	0.06±0.05	0	0	73±8	0	0	1±0.2	0	0
	M3	0.06±0.01	0	0	54±13	0	0	1±0.1	0	0
ZEN	L1	0	0	0	48±6	44±4	12±1	0	0	0
	L2	0	0.06±0.05	0	51±2	56±3	18±0.8	2±0.4	1±0.4	0.4±0.2
	L3	0.05±0.05	0.07±0.01	0.02±0.00	55±9	58±15	17±3	1±0.4	1±0.4	0.4±0.1
	M1	0	0	0	51±5	38±6	10±1	0	1±0.8	0.4±0.7
	M2	0	0.1±0.03	0	54±5	67±16	19±5	0.6±0.1	2±0.3	0.5±0.5
	M3	0	0.1±0.02	0.02±0.02	43±7	55±13	17±5	0.6±0.2	2±0.1	0.3±0.1
OTA	L1	0	0	0	52±4	0	0	0.8±0.1	0	0
	L2	0.05±0.01	0	0	55±13	0	0	0.7±0.2	0	0
	L3	0.06±0.03	0	0	56±9	0	0	0.5±0.05	0	0
	M1	0.08±0.1	0	0	41±5	0	0	0.3±0.4	0	0
	M2	0.08±0.03	0	0	61±8	0	0	0.9±0.1	0	0
	M3	0.07±0.01	0	0	45±7	0	0	0.8±0.2	0	0

Table S4. Instrumental MS/MS parameters of mycotoxins analysed in positive ionisation mode

Component	Rt (min)	Q1 (m/z)	Q3 (m/z)	DP (V)	EP (V)	CE (V)	CXP (V)
15-Acetyl-DON (qn)[1]	3.3	355.9	137.1	40	10	22	10
15-Acetyl-DON (ql)[2]	3.3	355.9	321.1	40	10	17	20
Aflatoxin B_1 (qn)	5.0	313.0	285.2	40	10	33	16
Aflatoxin B_1 (ql)	5.0	313.0	128.1	40	10	91	10
Deoxynivalenol (qn)	2.7	297.0	249.0	30	10	15	15
Deoxynivalenol (ql)	2.7	297.0	231.0	30	10	17	15
$^{13}C_3$-Caffeine (Internal standard)	3.3	198.0	140.0	40	10	29	12
Ochratoxin A (qn)	6.4	404.0	239.0	40	10	33	16
Ochratoxin A (ql)	6.4	404.0	102.0	40	10	91	14
Aflatoxicol (qn)	5.3	297.1	269.0	66	10	29	16
Aflatoxicol (ql)	5.3	297.1	114.9	66	10	81	12
Aflatoxin M_1 (qn)	4.1	328.9	272.9	61	10	33	18
Aflatoxin M_1 (ql)	4.1	328.9	229.0	61	10	55	16
Aflatoxin P_1 (qn)	4.3	299.0	271.0	101	10	33	18
Aflatoxin P_1 (ql)	4.3	299.0	114.9	101	10	71	12
Aflatoxin Q_1 (qn)	3.9	329.1	310.8	71	10	29	16
Aflatoxin Q_1 (ql)	3.9	329.1	177.0	71	10	45	22
$^{13}C_{17}$-Aflatoxin B_1	5.0	330.1	301.1	40	10	33	16
$^{13}C_{15}$-Deoxynivalenol	2.7	312.2	263.2	30	10	15	15
$^{13}C_{20}$-Ochratoxin A	6.4	424.2	250.0	40	10	33	16

[1] qn = quantifier ions [2] ql = qualifier ion

Table S5. Instrumental MS/MS parameters of mycotoxins analysed in negative ionisation mode.

Component	Rt (min)	Q1 (m/z)	Q3 (m/z)	DP (V)	EP (V)	CE (V)	CXP (V)
DON-3-glucoside (qn)[1]	2.7	517.2	457.1	-45	-10	-18	-13
DON-3-glucoside (ql)[2]	2.7	517.2	247.0	-45	-10	-32	-17
3-Acetyl-DON (qn)	3.4	397.1	337.0	-20	-10	-12	-23
3-Acetyl-DON (ql)	3.4	397.1	59.0	-20	-10	-52	-7
ß-Zearalenol (qn)	6.0	319.3	160.0	-110	-10	-41	-13
ß-Zearalenol (ql)	6.0	319.3	130.0	-110	-10	-47	-20
Zearalenone (qn)	6.5	317.1	175.0	-175	-10	-32	-15
Zearalenone (ql)	6.5	317.1	131.1	-175	-10	-36	-11
α-Zearalenol (qn)	6.6	319.2	160.0	-110	-10	-41	-13
α-Zearalenol (ql)	6.6	319.2	130.0	-110	-10	-47	-20
$^{13}C_{18}$-Zearalenone	6.5	335.2	185.1	-175	-10	-32	-15

[1] qn = quantifier ions [2] ql = qualifier ion

Table S6. Average recovery and within-lab reproducibility of mycotoxins in repeated analyses of a control sample (n=6)

**Component**	**Level (µg/kg)**	**Average recovery (%)**	**RSD_{wR}**
Aflatoxin B_1	5	100	2.8
Aflatoxicol	5	87	17
Aflatoxin M_1	5	91	12
Aflatoxin P_1	5	74	36
Aflatoxin Q_1	5	80	12
Deoxynivalenol	400	92	8.1
3-Acetyl-DON	200	95	22
15-Acetyl-DON	200	92	18
DON-3-glucoside	250	53	17
Zearalenone	100	106	4.7
α-Zearalenol	25	106	12
ß-Zearalenol	25	111	10
Ochratoxin A	2	103	20
